# Retraction: High-throughput metabolomics identifies serum metabolic signatures in acute kidney injury using LC-MS combined with pattern recognition approach

**DOI:** 10.1039/d1ra90062e

**Published:** 2021-02-01

**Authors:** Laura Fisher

**Affiliations:** Royal Society of Chemistry Thomas Graham House, Science Park, Milton Road Cambridge CB4 0WF UK advances-rsc@rsc.org

## Abstract

Retraction of ‘High-throughput metabolomics identifies serum metabolic signatures in acute kidney injury using LC-MS combined with pattern recognition approach’ by Hai-Hong Li *et al.*, *RSC Adv.*, 2018, **8**, 14838–14847, DOI: 10.1039/C8RA01749B

The Royal Society of Chemistry hereby wholly retracts this *RSC Advances* article due to concerns with the reliability of the data.

The images in the article were screened by an image integrity expert. Images published in Fig. 4 of the article have also been published in another article by a different set of authors.^1^

Furthermore, the left and right panels in Fig. 4A show duplicated images. In addition, the left and right panels in Fig. 4B are also identical.

The authors were unable to provide raw data for any of the images published in Fig. 4.

Given the significance of the concerns about the validity of the data, the findings presented in this paper are not reliable.

The authors do not agree with the retraction.

Signed: Laura Fisher, Executive Editor, *RSC Advances*

Date: 19^th^ January 2021

## Supplementary Material
